# Cell-Penetrating Protein/Corrole Nanoparticles

**DOI:** 10.1038/s41598-019-38592-w

**Published:** 2019-02-19

**Authors:** Matan Soll, Tridib K. Goswami, Qiu-Cheng Chen, Irena Saltsman, Ruijie D. Teo, Mona Shahgholi, Punnajit Lim, Angel J. Di Bilio, Sarah Cohen, John Termini, Harry B. Gray, Zeev Gross

**Affiliations:** 1 Schulich Faculty of Chemistry, Technion – Israel Institute of Technology, Haifa, 32000 Israel; 2 Department of Molecular Medicine, Beckman Research Institute of the City of Hope, Duarte, CA 91010 USA; 3 Beckman Institute, California Institute of Technology, Pasadena, CA 91125 USA

## Abstract

Recent work has highlighted the potential of metallocorroles as versatile platforms for the development of drugs and imaging agents, since the bioavailability, physicochemical properties and therapeutic activity can be dramatically altered by metal ion substitution and/or functional group replacement. Significant advances in cancer treatment and imaging have been reported based on work with a water-soluble bis-sulfonated gallium corrole in both cellular and rodent-based models. We now show that cytotoxicities increase in the order Ga < Fe < Al < Mn < Sb < Au for bis-sulfonated corroles; and, importantly, that they correlate with metallocorrole affinities for very low density lipoprotein (VLDL), the main carrier of lipophilic drugs. As chemotherapeutic potential is predicted to be enhanced by increased lipophilicity, we have developed a novel method for the preparation of cell-penetrating lipophilic metallocorrole/serum-protein nanoparticles (NPs). Cryo-TEM revealed an average core metallocorrole particle size of 32 nm, with protein tendrils extending from the core (conjugate size is ~100 nm). Optical imaging of DU-145 prostate cancer cells treated with corrole NPs (≤100 nM) revealed fast cellular uptake, very slow release, and distribution into the endoplasmic reticulum (ER) and lysosomes. The physical properties of corrole NPs prepared in combination with transferrin and albumin were alike, but the former were internalized to a greater extent by the transferrin-receptor-rich DU-145 cells. Our method of preparation of corrole/protein NPs may be generalizable to many bioactive hydrophobic molecules to enhance their bioavailability and target affinity.

## Introduction

Porphyrins and their metal complexes (metalloporphyrins) have been explored for their anticancer applications ever since they were first proposed for cancer diagnosis by Figge over 75 years ago^1^. Fast forwarding to more recent times, many investigators have found that these molecules are versatile theranostic agents adaptable to multiple imaging methodologies (e.g., optical, magnetic resonance, ratiometric)^2,3^ in combination with cell killing ability initiated by an exogenous stimulus such as light or ultrasound^4–7^. A key finding from these studies is that selectivity for cancerous *vs*. healthy tissue and preferential cellular uptake by the former often depend on porphyrin association with either native or synthetic carrier proteins^8–10^. In addition, many of these investigations have relied on overexpression of specific receptors to enable cancer cell specific targeting^11–13^. Of relevance here is that the search for more efficacious imaging and therapeutic agents has been extended to include expanded and contracted porphyrins^14–16^.

**Figure 1:**
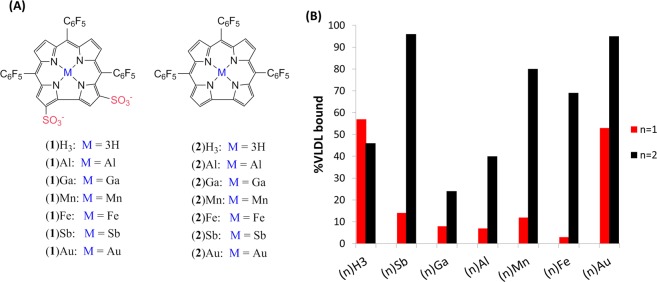
(**A**) Molecular structures of the investigated corroles. (**B**) Selectivity of (**1**)M (red bars) and (**2**)M (black bars) complexes to the VLDL fraction of human serum.

Among the family of contracted porphyrins, our focus has been on the water-soluble metallocorroles; and, significantly, we have found that these molecules are very promising drug candidates for both the prevention (diabetes, heart and neurodegenerative) and treatment (neurorescue and cancer) of diverse diseases^17–23^. Control of desired biological and biophysical properties has been governed mainly by the identity of the corrole-chelated metal ion, while cells/tissue affinity can be directed by substituents on the corrole periphery. Selectivity for specific cancers has been achieved via conjugation of amphipolar metallocorroles, containing hydrophilic –SO_3_^−^ head groups on one pole of the otherwise lipophilic molecule (the (**1**)M complexes; Fig. 1A) to tumor-targeting proteins^24^. In parallel, different metal and functional group combinations have been examined in the absence of a carrier protein. These studies revealed that: (a) gold substituted corrole (**1**)Au is a much more potent anticancer agent than gallium corrole (**1**)Ga; (b) most of the activity is attributable to late cell cycle arrest; and (c) increasing the lipophilicity (e.g., replacing two sulfonic acid head groups with one carboxylic acid) results in improved cytotoxicity^25–27^.

## Results and Discussion

We began by investigating the influence of the metal ion chelated by the amphipolar corrole (**1**)H_3_; by comparing the cytotoxicity of six (**1**)M complexes (M = Au, Al, Ga, Sb, Mn, or Fe) against four cancer cell lines (Fig. 1A, Table 1). Interestingly, the gold(III) complex (**1**)Au was invariably the most cytotoxic, with 2–10 times lower IC_50_ values relative to the other metallocorroles. As the chelated element in the otherwise identical complexes might affect lipophilicity or association with serum proteins^28^, we determined the octanol/water partition coefficient (log*P*_ow_) and the affinity to particular serum fractions for the entire series. We found some variability in the log*P*_ow_ values, but no correlation with IC_50_ values. On the other hand, cytotoxicity was clearly enhanced for metallocorroles with the highest affinity for the very-low-density lipoprotein fraction (VLDL) of human serum: (**1**)Mn, (**1**)Sb, and most significantly (**1**)Au. The latter had the highest affinity by far (53% vs. 3–14%) for VLDL and IC_50_ values that were 2–10 times lower than those of all other complexes. As VLDL is the primary carrier of lipophilic compounds^29,30^, and considering that recent work has shown that it may be exploited to enhance lipophilic drug uptake via the LDL receptor^30^, we reasoned that enhanced VLDL loading could be attained by increasing corrole lipophilicity. Our focus turned to the much more lipophilic (**2**)M metallocorroles, where we found that VLDL affinity was significantly enhanced, ranging from 24% for (**2**)Ga to almost 100% for (**2**)Sb and (**2**)Au (Fig. 1B). These experiments highlighted the solubility problem, as especially evident in 10% DMSO/PBS solutions: precipitation was observed at ≥100 μM corrole; while NPs formed in the 10–1 μM corrole range. We further found that even the much more water-soluble (**1**)Ga forms NPs when combined with cancer-targeting proteins^24^. Following that work, we set out to conjugate lipophilic metallocorrole [(**2**)M] NPs with *native* serum proteins in the hope that these assemblies would be soluble in aqueous media. We initially focused on albumin (both bovine, BSA, and human, HSA), because of their abundance, stability and high solubility.

**Table 1:** Cytotoxicity of (**1**)M complexes to cancer cells, their lipophilicity, and their partitioning between human serum lipoproteins.

Compound	Cytotoxicity (IC_50_, μM)				log*P*_ow_^a^	Distribution in human serum (%)^b^		
	DU 145	SK-MEL-28	MDA-MB-231	OVCAR-3		VLDL	LDL	HDL
(**1**)Au	48	27	20	28	0.55	53	5	42
(**1**)Sb	90	88	78	51	0.37	14	12	74
(**1**)Mn	224	98	38	45	0.21	12	7	81
(**1**)Al	156	98	82	151	0.40	7	8	85
(**1**)Fe	173	124	109	188	0.55	3	4	93
(**1**)Ga	159	131	129	274	0.72	8	10	81

^a^Octanol/water partition coefficient. ^b^VLDL, LDL, and HDL, are (very-low)-, low- and high-density lipoproteins.

Albumin-based NPs as controlled release drug delivery systems have earlier been constructed using a variety of nanotechnology techniques such as desolvation, emulsification, thermal gelation, nano-spray drying, and self-assembly, and their chemicophysical properties have been widely investigated^31–39^. Examination of the relevant literature revealed that the vast majority of NPs used for drug delivery required modifications of the albumin macromolecule; and, in the case of porphyrin packaging, the preparation of special derivatives was essential. Of the numerous reports describing the formulation of porphyrin loaded NPs^38–43^, we were interested mainly in those using tetraaryl derivatives which obviated the requirement for surfactants in their assembly. A prominent example was reported in 2002 by Gong and coworkers^40^, where addition of water to DMSO solutions of porphyrin initiated the formation of NPs in the presence of polyethyleneglycol (PEG). Employing a different procedure, Chen and coworkers loaded porphyrins onto preformed albumin NPs, obtained by desolvation and glutaraldehyde cross linking^38,39^. In contrast, in our work we decided to rely on unmodified proteins for the stabilization and solubilization of NPs formed by lipophilic corroles in aqueous media, based on our previous observation of the facile formation of NPs from water-soluble (**1**)Ga and (**1**)Mn with a semi-synthetic protein^24^.

**Figure 2:**
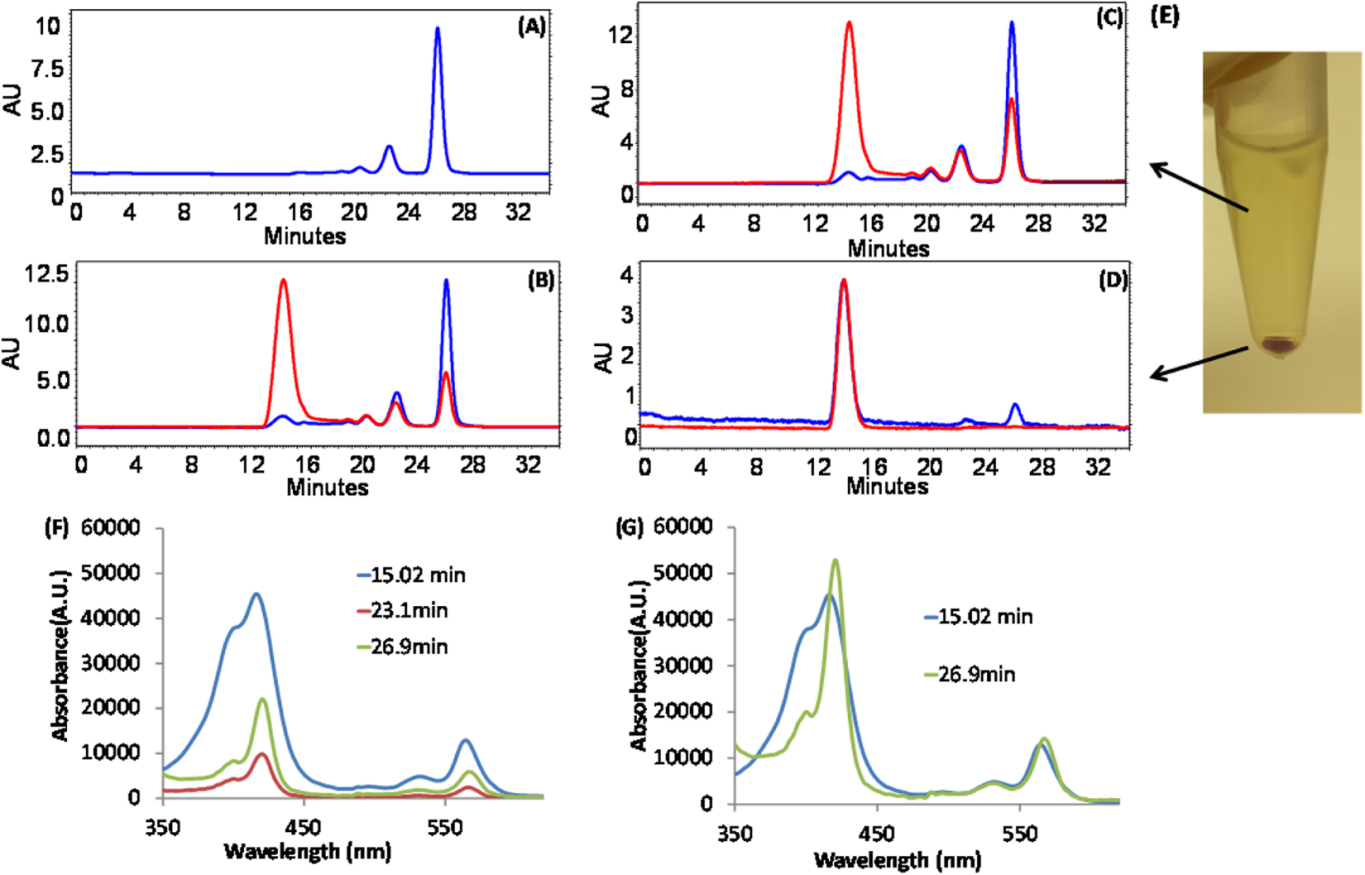
HPLC chromatograms (Sephadex TM 200 10/300 GL column) with recording at 280 nm (blue line) and 416 nm (red line) for detection of BSA and corrole, respectively, performed for (**A**) untreated BSA; (**B**) the (**2**)Au/BSA conjugate before centrifugation; (**C**) the mother liquor of the (**2**)Au/BSA conjugate after mild centrifugation (5,000x g for 30 min); and (**D**) the (**2**)Au/BSA precipitate obtained upon centrifugation and re-solvation. (**E**) NPs obtained from the (**2**)Au/BSA conjugate after centrifugation at 20,000 x g for 30 min; (**F**) UV-vis spectra recorded for the HPLC fractions obtained for the (**2**)Au/BSA conjugate before centrifugation at three relevant elution times; and (**G**) normalized (to the Q band at 564 nm) UV-vis spectra recorded at the indicated elution times.

The methodology that we report here consists of a mixed-solvent medium that dissolves both the lipophilic metallocorrole and the protein, followed by sequential dialysis to remove the organic solvent. This procedure was employed for all six (**2**)M complexes, yielding strongly colored 0.1 mM aqueous solutions *free of organic solvent*. The corrole/albumin conjugates were characterized by size exclusion HPLC, UV-vis and circular dichroism (CD) spectroscopy, dynamic light scattering (DLS), and scanning electron microscopy (SEM). The chromatographic profile shown in Fig. 2 for the combination of (**2**)Au and BSA was typical of that observed for all (**2**)M corroles formulated with albumin (SI, Figs S1–S2), i.e., a very high molecular weight corrole/albumin fraction containing more corrole than albumin and eluting at ~15 min, followed by a much later eluting (~27 min) corrole-free albumin peak (Fig. 2B). Importantly, the 15-min peak was not observed upon identical treatment of BSA with DMSO in the absence of corrole (Fig. 2A). UV-vis analyses confirmed that the vast majority of corroles appeared in the early eluting fraction. Furthermore, while there were no major shifts in the λ_max_ values, there was substantial broadening of the near-UV absorption (the Soret band) in the early eluting fraction (15 min) relative to late eluting fractions (23 and 27 min) (Figs 2F,G, S2).

A Bradford standard test indicated that protein content in NPs prepared from an initial 10:1 BSA/(**2**)Ga ratio was <2% of the total albumin protein weight, while UV-vis estimation of encapsulated corrole concentration indicated that the albumin/corrole ratio was ~1:8. Lyophilized conjugates were stable for months at 4 °C, and their re-suspended solutions remained stable for several weeks. Simple centrifugation led to separation of conjugates from excess albumin, as confirmed by HPLC (Fig. 2C–E), and the process of re-suspension and centrifugation provided strong evidence for NP formation. However, the isolated material was less stable, becoming insoluble after several cycles, thereby indicating that an excess of free albumin enhances NP solubility.

**Figure 3:**
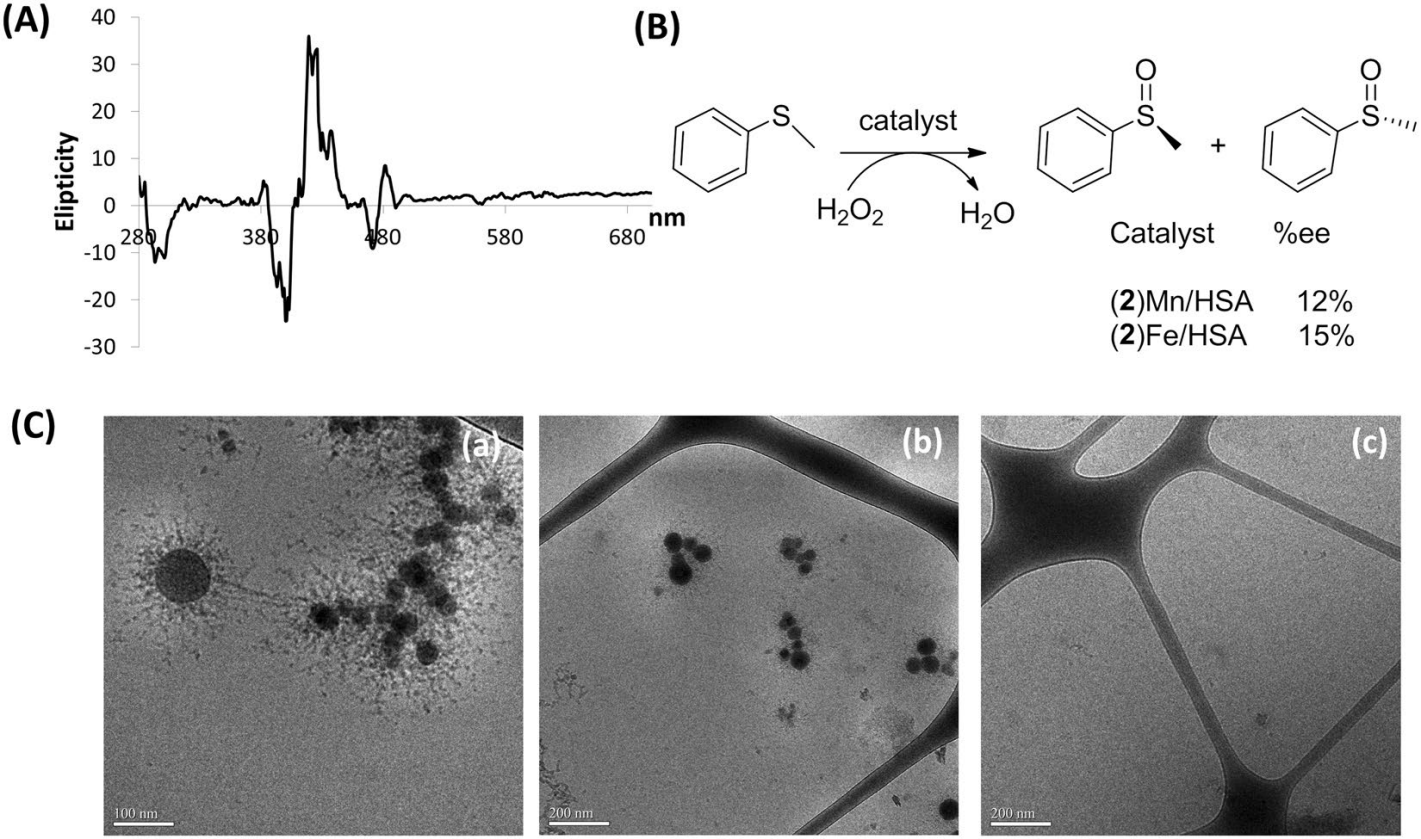
(**A**) The induced CD spectrum recorded for (**2**)Mn/BSA NPs and (**B**) asymmetric oxidation of thioanisole by H_2_O_2_ in solutions that contained NPs composed of HSA with Fe(III)/Mn(III)corroles. (**C**) Cryo-TEM images of (**2**)Au/BSA (a and b, at two different magnifications) and of similarly treated BSA but without corrole (c). Scale bars represent 100 nm (a), 200 nm (b), and 200 nm (c).

The structure of the albumin/corrole NPs was first examined both by low resolution scanning electron microscopy (SEM) and atomic force microscopy (AFM), as illustrated for an isolated dry (**2**)Au/BSA pellet on a silicon wafer (Fig. S3). The SEM-estimated size was in the range of 150 ± 20 nm; however, the AFM-based estimation of the distance from the surface (<20 nm in height) was more consistent with an ovoid flattened disc than a regular sphere. Importantly, similarly treated BSA in the absence of corrole did not exhibit any geometrically ordered structure, as there were only disordered aggregates resulting from centrifugation. Dynamic light scattering of isolated and re-suspended (**2**)Au-based NPs in PBS indicated a wide distribution of particle sizes, with an average of 180 ± 84 nm (Fig. S3). The apparent contradiction between size estimates obtained for solid and solvated states was resolved by cryogenic transmission electron microscopy (cryo-TEM) of NPs in solution (Fig. 3C). The core size was found to be <100 nm, with an average of 32 ± 12 nm (Fig. S11), while hydrophilic protein extensions increased it to ≥100 nm. We therefore conclude that corrole/protein assemblies consist of spheroidal corrole-NP hydrophobic cores that are stabilized and water solubilized by noncovalently attached proteins. Importantly, this sea urchin-like structure with a high surface area to volume ratio likely facilitates endocytosis and uptake across a variety of cell lines.

**Figure 4:**
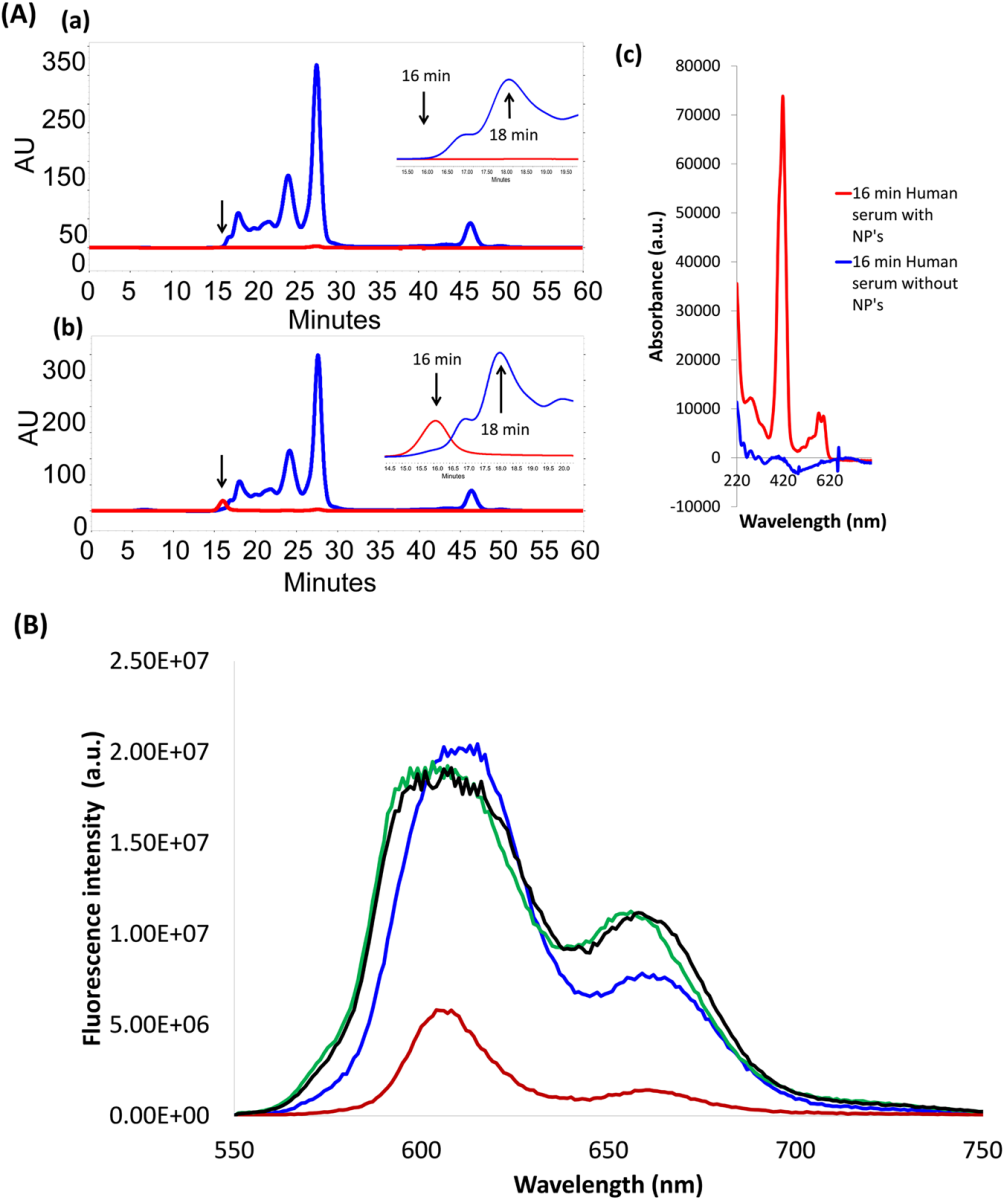
(**A**) HPLC traces, with reading set at 280 nm (blue line) for detection of lipoproteins and at 420 nm (red line) for detection of corrole, of human serum samples incubated for 2 h without (a) and with (b) (2)Ga/BSA based NPs; and the UV-vis spectra recorded (c) for the 16-min-eluting fraction of both samples. (**B**) Effect of encapsulation on the emission of virtually identical concentrations of (**2**)Ga in DMSO (blue trace), dialyzed NPs formed with BSA (red trace), treatment of the latter solution with 2% Triton-X (green trace), and extraction of (**2**)Ga from the last solution into dichloromethane (black trace). Note that instrumental parameters were the same in all cases.

Additional information about NP constitution was obtained from CD analyses, deployment as asymmetric catalysts, and mass spectroscopy (Figs 3A,B, S5 and S8). Strong exciton CD coupling in the visible region corresponding to absorption of non-chiral (**2**)Mn is consistent with its intimate association within the chiral environment of albumin (Fig. 3A). Close association of metallocorroles with the chiral protein also was indicated by modest enantioselectivity in the H_2_O_2_ oxidation of thioanisole using albumin NP catalysts containing either (**2**)Mn or (**2**)Fe (Fig. 3B, Table S2). In order to investigate whether covalent bonds might form during NP assembly, possibly via substitution of C_6_F_5_ *para*-F atoms by nucleophilic amino acids in albumin^44,45^, several methods were employed to discriminate between non-covalent and covalent associations. While we were unable to extract (**2**)Al into the organic phase following prolonged (24 h) stirring of (**2**)Al-based NPs in a PBS/CH_2_Cl_2_ solvent system, denaturation of the NP-bound protein with 2% Triton-X led to solubilization of (**2**)Al in the organic phase (Fig. S6). Spectroscopic analysis of recovered corrole (Fig. S7b,c) revealed no structural changes in the macrocyclic periphery (^1^H NMR, β-pyrrole CH protons) or the C_6_F_5_ groups (^19^F NMR), thereby indicating that tight but non-covalent association of the corrole NP with albumin occurred. Examination of NPs by MALDI MS provided additional evidence for noncovalent binding, as only signals corresponding separately to albumin and (**2**)Ga (Fig. S8) were observed. Compelling evidence for corrole aggregation within the assembly was obtained by comparing the emission spectra of (**2**)Ga-based NPs with virtually identical concentrations of the same corrole under conditions where no NPs were formed (Fig. 4B). Utilizing the same amount of a DMSO stock solution of (**2**)Ga for dilution into either DMSO (blue trace) or BSA-containing PBS buffer (red trace), we found that fluorescence in aqueous media was strongly quenched. Full recovery of emission was however observed upon treatment with Triton-X above its critical micelle concentration, in both the aqueous medium (green trace) and when the monomerized (**2**)Ga was extracted into an organic solvent (the black trace).

The interactions of metallocorrole/protein conjugates with proteins present in serum were investigated not only as the first step toward utilization, but also because the HPLC elution times of the preformed NPs were suspiciously similar to that of VLDL. The results obtained upon 2 h incubation of (**2**)Ga/BSA NPs with human serum clearly show that they elute earlier than VLDL; in addition, no serum fraction was found with corrole released from the initial assembly (Fig. 4A). It is likely, then, that metallocorrole cargo will not be released rapidly during circulation (which in turn suggests that such cargo may not be released from (**2**)Ga/BSA NPs upon *in vivo* application). The key question now is: could such NP-protein conjugates enhance cellular uptake; and, if they do, could this approach be used for targeting specific cells?

**Figure 5:**
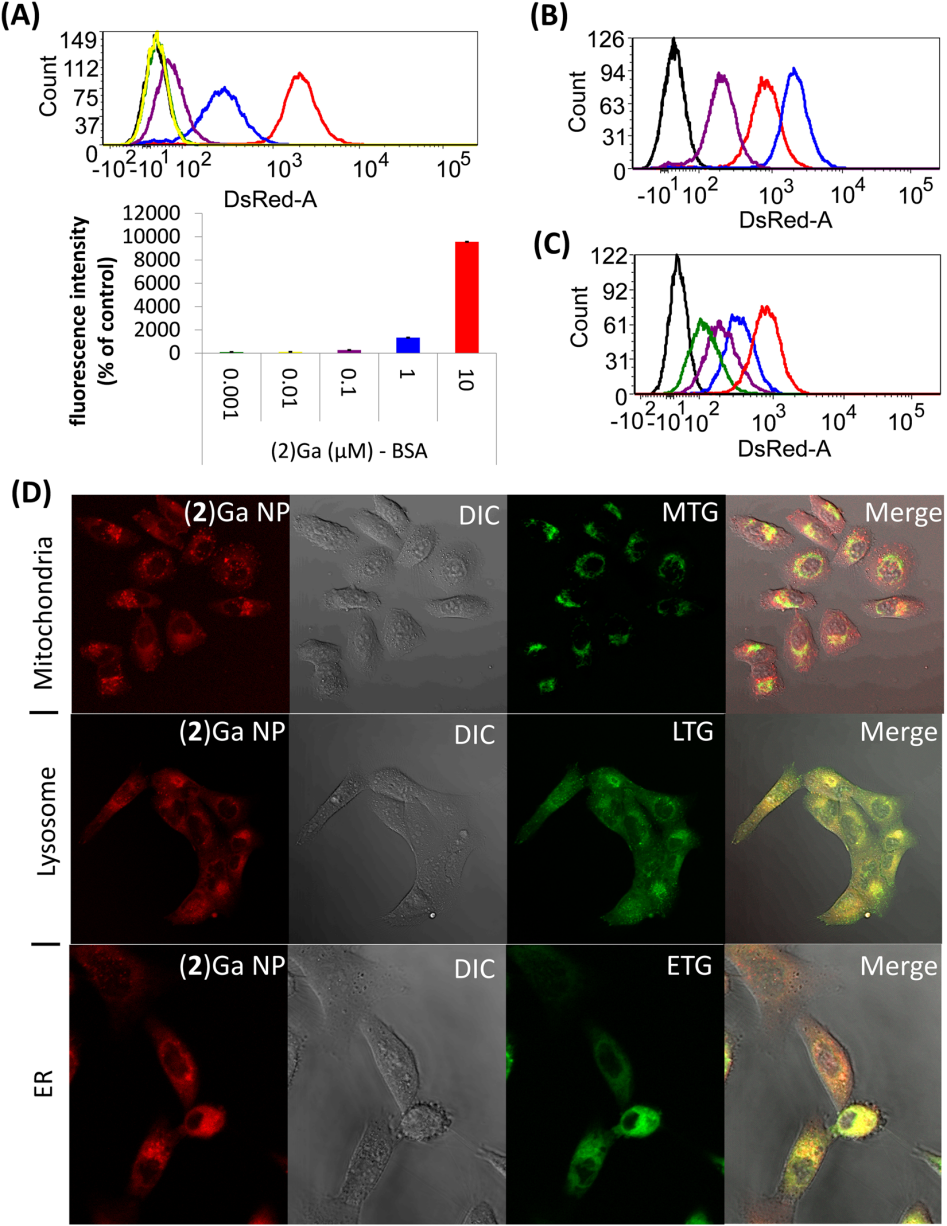
Intracellular uptake, clearance, and localization of (**2**)Ga NPs. (**A**) Dose dependent uptake of (**2**)Ga NPs by prostate cancer cells (DU-145) measured by FACS following 24 h incubation with (**2**)Ga NPs at corrole concentrations of 10 μM (red line), 1 μM (blue line), 0.1 μM (purple line), 0.01 μM (yellow line), 0.001 μM (green line), and with BSA as control (black line). Quantification of relative median fluorescence intensities displayed as percentage of fluorescence intensities relative to the control. (**B**) Uptake of (**2**)Ga NPs (10 μM) in DU-145 cells measured at incubation times of 2 h (purple line), 8 h (red line) and 24 h (blue line). (**C**) Clearance of (**2**)Ga NPs (10 μM) from DU-145 cells portrayed by the loss of fluorescence intensities beginning from t = 0 (red line; florescence after 24 h incubation and replacement by NP-free medium) through 24 h (blue line), 48 h (purple line) and 72 h (green line). (**D**) Immunofluorescence co-localization of (**2**)Ga NPs (red) and various organellar markers (green). Live imaging of DU-145 cells incubated with (**2**)Ga NPs (10 μM) for 24 h and stained for mitochondria (Mitotracker green; MTG), lysosome (lysotracker green; LTG) or endoplasmic reticulum (ER-tracker green; ETG). Fluorescence was recorded at 40x magnification on a LSM700 confocal system supported with Zen software. Representative images of 12 separate fields.

Cellular uptake was evaluated by optical imaging of NPs containing fluorescent corroles. Specifically, we investigated time- and dose-dependent incubation of (**2**)Ga–based NPs with prostate cancer human cells (DU-145). Intracellular fluorescence was easily detectable after 24 h for cells treated with nanomolar concentrations of corrole (Fig. 5A), and it persists even after 72 h of incubation of the cells with corrole-free medium (Fig. 5B). The time dependent loss of fluorescence is in fact so slow that it might be attributed to dilution owing to cellular division rather than to (**2**)Ga diffusion into the medium. The ability to detect very low concentrations of (**2**)Ga, whose emission is strongly quenched within the NPs (*vide supra*), further suggested that the images might include corroles that had become less aggregated upon internalization into the protein-rich cellular fluid. In turn, this likely outcome led us to investigate corrole intracellular distribution, which we evaluated with the aid of molecular organelle markers specific for mitochondria, the endoplasmic reticulum (ER), and lysosomes. Fluorescent tags that emit green light were used in all cases, as these allow very sensitive detection of red-emitting corroles. The results (Fig. 5D) clearly show that (**2**)Ga accumulates mainly in the ER and lysosomes, with at most very small amounts in mitochondria.

**Figure 6:**
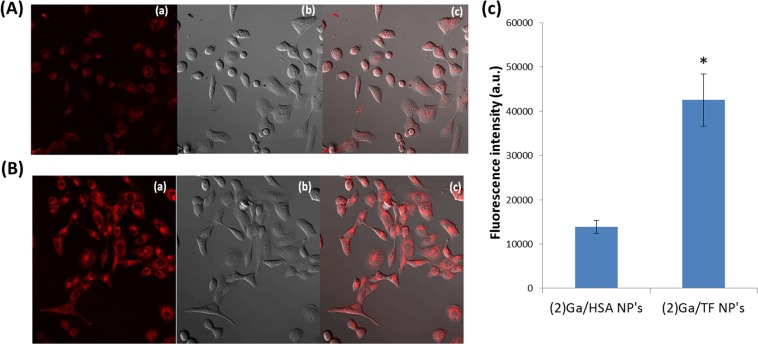
Live imaging of DU-145 cell line after 24 h incubation with 10 μM of (**A**) (**2**)Ga/HSA NPs and (**B**) (**2**)Ga/TF NPs: (a) under conditions specific for detection of (**2**)Ga fluorescence (excitation at 405 nm); (b) phase contrast microscopy; and (c) superimposed image of (a) and (b). Fluorescence was recorded using a 40x objective and a LSM700 confocal system supported with Zen software. (**C**) Quantification of fluorescent signals from 12 separate fields. Data are expressed as mean ± SEM n = 4; *p < 0.01 vs. (**2**)Ga/HSA NP’s treated cells.

An important step toward extension of our methodology was the finding that corrole NPs also can be prepared with apo-transferrin (TF) rather than with albumin, under virtually identical conditions. In work that followed, we compared the cellular uptake of NPs prepared by combining (**2**)Ga with each of these proteins. The much more intense fluorescence recorded for (**2**)Ga/TF relative to (**2**)Ga/HSA (Fig. 6) clearly indicates greater internalization of the transferrin-covered NP. Considering that transferrin receptors are overexpressed in the human prostate cancer cell line DU-145 used in our study^46^, this finding could have been anticipated if we had been confident that the NPs would not have modified the associated proteins during treatment. *Apparently, they did not!*

In summary, we have developed a user friendly method for the synthesis of metallocorrole/protein nanoparticles. Formulation of NPs using specific proteins will enable targeting to cells in which the corresponding receptors are overexpressed. Most importantly, corroles released from NP conjugates can be used for multiple purposes, including optical imaging and therapeutic targeting. The ongoing focus is on determining the factors that govern NP sizes and structures as well as their relationships to rates of cellular uptake and other bioactivities that could lead to game changing advancements in both detection and selective destruction of cancerous tissues. The mechanism(s) of cellular uptake and the influence of NP size will be important to determine, as it is predicted to influence both the uptake kinetics and the intracellular half-life. For example, for imaging applications, prolonging the intracellular half-life would be desireable, while this would be less advantageous for therapeutic applications. This can be regulated by controlling the NP size for specific applications. The transferrin formulated NPs showed enhanced uptake for transferrin receptor-rich DU-145 cells relative to HSA NPs, suggesting that the transferrin components of the NPs underwent minimal exchange with more abundant serum components prior to internalization. The NPs may have been held together by a corrole scaffold which stabilized them towards exchange, but verification of this possibility awaits additional structural studies on the supramolecular assembly. The stability and enhanced uptake properties of the transferrin NPs may find utility for the targeting of lipophilic cargo to the brain, given the abundance of transferrin receptors in the blood brain barrier. The potential for imaging applications using Mn(III) corroles as MRI contrast enhancing agents, or other suitable metal-substituted corroles for NIR and optical imaging in the brain, opens up many avenues of exploration for studies of both primary and metastatic brain tumors, which have been heretofore limited by the well known neurotoxicity of the widely used gadolinium imaging agents.

## Methods

### Chemicals and reagents

Media, serum, antibiotics, and supplements were purchased from Biological Industries (Beit Haémek, Israel). Mitotracker Green (MTG), lysotracker green(LTG) and ER-tracker green (ETG) were obtained from Life Technologies, Rhenium (Jerusalem, Israel). The materials used for synthesis and work-up procedures were purchased from Sigma Aldrich, Merck, Fluka and Frutarom and used as received unless otherwise stated. Deuterated solvents (Sigma Aldrich isotopes products) with a 99.5% minimum deuteration were used as received. Silica gel for column chromatography (Silica Gel 60, 63–200 µm mesh) was obtained from E. Merck Ltd. Pyrrole was run through a short basic alumina column and aldehydes were purified by vacuum distillation before use.

### Synthetic procedures

Metallocorroles used for albumin conjugation from the (**1**)M and (**2**)M series were prepared by previously reported procedures^23,47–59^.

### Formulation of albumin/corrole nanoparticles

A solution of 400 µL of 1 mM corrole dissolved in DMSO was added dropwise (0.04 mL/min) to a 3.6 mL of 100 µM BSA or HSA in PBS pH 7.2 stirred at 500 rpm in a 5 °C water bath. Solutions were incubated for 30 min at 5 °C and transferred to dialysis tubing for 24 h dialysis in a 1 liter PBS solution. Dialysis tubes were purchased from Spectrumlabs cat. 3787-D20; type: RC; MWCO: 12–14000. Dialysis tubing was treated vigorously with EDTA: tubing was immersed into 1 L 2% sodium bicarbonate/1 mM EDTA in a 2 L glass beaker. Tubing was rinsed thoroughly with ddH_2_O (sterile ultra-pure water) and submerged completely in 50% Ethanol/1 mM EDTA and stored at 4 °C. Tubing was rinsed thoroughly before use.

Optional: Samples of NP’s in PBS could be freeze dried for 24 h in a −50 °C lyophilizer to dryness. Dried conjugates could be re-suspended in 4 mL PBS in order to yield a 1 mM corrole solution. Running of the corrole samples in PBS in a S300 sapharose gel column yielded a signal in the range of 420 nm for the corrole at a retention time of 15 min only, whereas reading at the 280 nm for albumin yielded a signal at the retention time of 15 min (smaller) and 35 min. The eluent was PBS and the flow rate was set at 0.5 mL/min. The products of the lyophilized conjugates are dry albumin and nanoparticles ranging in sizes of <100 nm. The product was stable as solid for months under −20 °C storage, while re-suspended solutions were stable for a few weeks.

### SEM analysis

100 µL of NP samples were placed on silicon wafers and left inside a chemical hood to fully dry. Silicon wafers were then taken to be coated with gold using Polaron Sputter coater. Samples were measured by TESCAN (Vega-II) Scanning Electron Microscopy supported by TESCAN software system.

### AFM analysis

100 µL of NP samples were placed on silicon wafers and left inside a chemical hood to fully dry. Samples were than measured using a Veeco (Dimension 3100) Atomic Force Microscope (AFM) operated by a NanoScope IIIa Controller.

### DLS and Nanosight NS300 systems

Samples were diluted by a factor of 10,000 in PBS. They were measured using the Nanosight NS300 or the PSS Nicomp 380 DLS-ZLS Analyzer in accordance with manufacturer’s instructions.

### 1.3.5 cryo-TEM analysis

Samples were prepared to be at a mass percentage of 1% particles in PBS and the Cryo-TEM specimens were prepared in a controlled environment vitrification system (CEVS). Cryogenic transmission electron microscopy (cryo-TEM) imaging was performed either by a Phillips CM120 or a FEI Talos 200 C, FEG-equipped cryo-dedicated high-resolution transmission electron microscope (TEM and STEM), operated at an accelerating voltage of 120 kV. Specimens were transferred into an Oxford CT-3500 cryo-holder (Philips) or a Gatan 626DH (FEI) cryo-holder, and equilibrated below −178 °C. Specimens were examined using a low-dose imaging procedure to minimize electron-beam radiation damage. Images were recorded digitally by a Gatan Multiscan 791 cooled CCD camera (Philips CM 120), or a Gatan US 1000 high-resolution CCD camera (Tecnai T12 G2), using DigitalMicrograph software.

### HPLC

HPLC analysis was performed using a MERCK HITACHI HPLC system with a diode array detector supported with HPLC Chromaster Driver for Waters® Empower™3 Software. 10 µL of each sample were injected using the auto sampler. Size exclusion chromatography was done with either a SuperoseTM 6 10/300 GL gel column or a sephadexTM 200 10/300 GL column (as noted), with 0.5 mL/min eluting rate and sterilized PBS (Sigma, sterile-filtered, isotonic, pH 7.2) as eluent.

### Asymmetric oxidation of thioanisole by H_2_O_2_

The catalytic reaction was carried out at room temperature, overnight, in PBS pH7. The ratio of reagents was: oxidant (3% H_2_O_2_):substrate:serum albumin:catalyst (0.2 mM) 75:50:1.5:1 respectively.

### GC

The analysis of the catalytic reaction products, sulfoxidation of thioanisole, was performed on a GC consisting at a Sion 4210 GC System and equipped with chiral capillary column Astec CHIRALDEX B-PM (L × I.D. 30 m × 0,26 mm, df = 0.12 μm).

### Human cancer cell lines

One cell line from the NCI60 cell panel was used in this study: DU-145 (prostate cancer). Cells were grown in EMEM cell culture medium (ATCC) containing 2mM L-glutamine, supplemented with 10% FBS (Biological Industries), and maintained at 37 °C under 5% CO_2_ in a humidified incubator.

### FACS analysis, general procedure

Cells (DU-145) were seeded in 6-well microtiter plates (5 × 104 cells/mL; 3 mL per well) 24 h before the addition of assigned compounds/albumin particles. At the time of drug treatment, stock solutions of compounds were diluted to 10-fold the desired final test concentrations with EMEM medium. Aliquots of 300 μL of these diluted solutions were added to the appropriate microtiter wells containing 2700 μL of medium, resulting in the required final drug concentrations (according to assigned concentration). The final concentration of DMSO (given that the compound required DMSO for solvation) in test culture was <1%. All cells were incubated in the dark throughout the 24–72 h incubation period and were not exposed to light for prolonged periods. Following 24–72 h of incubation at 37 °C, cells were dissociated with trypsin, re-suspended in PBS pH 7.2, and pelleted at 3000 rpm for 5 min. Samples were washed 3 times with PBS, after which they were taken for measurement using a BD LSR-II Analyzer supported with BD FACSDiVaTM Software Version 6.1 for data analysis. Corrole fluorescence was measured using appropriate filters for excitation at 405 nm and reading emission at 660/20 nm.

### Live cell imaging, general procedure

Cells (DU-145) were seeded in a 24-well microtiter plates with a glass optic bottom (5 × 104 cells/mL; 0.9 mL per well) 24 h before the addition of assigned compounds/NPs. At the time of drug treatment, stock solutions of compounds were diluted to 10-fold the desired final test concentrations with EMEM medium. Aliquots of 100 μL of these diluted solutions were added to the appropriate microtiter wells containing 900 μL of medium, resulting in the required final drug concentrations (according to assigned concentration). The final concentration of DMSO (given that the compound required DMSO for solvation) in test culture was <1%. All cells were incubated in the dark throughout the 24 h incubation period and were not exposed to light for prolonged periods. Following 24 h of incubation at 37 °C, Mitotracker Green (MTG), lysotracker green(LTG) and ER-tracker green(ETG) (Life Technologies, Rhenium, Jerusalem, Israel) were added in accordance with manufacturers instructions and were optimized for best assigned concentrations/incubation time and tagging results. Cells were treated with MTG (200 nM, 30 min), LTG (150 nM, 2 h) and ETG (2 μM, 2 h). Corrole uptake and organelle tagging was measured using a 40x objective and a LSM700 confocal system supported with Zen software. Samples were excited at 488 nm for MTG, LTG and ETG detection (3%, 5% and 5% respectively); and at 405 nm (10%) for (**2**)Ga detection.

### Separation of corrole-containing serum

500 µL of aqueous solutions containing 200 µM corrole were added to 500 µL of human serum and co-incubated at room temperature for at least 15 minutes prior to further treatment. The solutions were filtered through a 0.22 μm filter and 50 μL were injected to a LaChrom Elite HPLC system fitted with a superose 6 10/300 GL (GE healthcare) gel filtration column and a photodiode array detector. The samples were eluted with PBS (pH 7.2) at a flow rate of 0.5 mL/minute. Chromatograms at 280 nm and at the λ_max_ of each corrole (424, 433, 426, 420, 424, 420 and 426 nm for (1/2)H_3_, (1/2)Au,(1/2)Sb, (1/2)Mn, (1/2)Al, (1/2)Fe and (1/2)Ga respectively) were recorded. The electronic spectra were recorded at time frames where the eluted corrole-containing lipoproteins were maximal.

### Statistical analysis

Data were expressed as the mean ± S.E.M and were compared between experimental groups with the use of one-way analysis of variance followed by Tukey’s post hoc test unless otherwise specified (Analyze-it software for Windows Excel, Leeds, UK). Probability values of p < 0.05 were considered to be statistically significant.

### NMR

The ¹H NMR and ^19^F NMR spectra were recorded on Bruker AM 200 and AM 300, operating at 200 and 300 MHz for 1 H and 188 MHz for 19 F (on AM 200), respectively. Chemical shifts are reported in ppm relative to residual hydrogen atoms in the deuterated solvents: 7.24 for chloroform.

## Supplementary information

SI
