# Supplementary material for: Cell-Penetrating Protein/Corrole Nanoparticles
Source: Sci Rep. 2019 Feb 19;9:2294. doi: 10.1038/s41598-019-38592-w (PMC6381154; doi:10.1038/s41598-019-38592-w)
Supplement: Supplementary file 1 — SI [file 41598_2019_38592_MOESM1_ESM.pdf]

Supporting Information

Cell-Penetrating Protein/Corrole Nanoparticles

Matan Soll, [a] *Tridib Goswami,* [a] *Qiu-Cheng Chen,* [a] *Irena Saltsman,* [a] *Ruijie D. Teo,* [c] *Mona Shahgholi,* [c] *Punnajit Lim,* [b] *Angel J. Di Bilio,* [c] *Sarah Cohen,* [a] *John Termini,* *[b] *Harry B. Gray**[c] *and Zeev Gross**[a]

a Schulich Faculty of Chemistry, Technion – Israel Institute of Technology, Haifa 32000, Israel

b Department of Molecular Medicine, Beckman Research Institute of the City of Hope, Duarte, CA 91010

c Beckman Institute, California Institute of Technology, Pasadena, CA 91125

Table of contents

Figure S1. Chromatograms for detection of BSA and the corroles 3
Figure S2. Spectra recorded for the HPLC-injected corrole/BSA conjugates 4
Figure S3. Imaging of nanoparticles and estimation of size 5
Figure S4. Solubility of formulated biconjugated corrole NP's 6
Figure S5. CD spectra of the different corrole NP's.. 7
Figure S6. (2)Al conjugated to BSA NPs in PBS after extraction..................................... 8
Figure S7. 19F NMR in D2O (400Mhz) of (2)Al/BSA NP's and 19F/1H NMR after extraction.. 8
Figure S8. MALDI-mass spec of (2)Ga BSA NP's 9
Figure S9. Quenching of the fluorescence of (2)Ga upon encapsulation in albumin based NP's.. 9
Figure S10. Chromatograms for detection of corrole/Ts NP's and DLS analysis 10
Figure S11. Particle size distrebution by Cryo-TEM imaging 11
Table S1. IC50 values of (2)M series in DMSO(aq.) 0.1% 11
Table S2. Asymmetric oxidation of thioanisole by H2O2 catalysed by either HSA, BSA, or NPs... 12
Figure S12. HPLC traces of human serum incubated with the corresponding corroles ... 13
Figure S13. Cytotoxicity graphs of (1)M series.. 14
Figure S14. Percentage binding affinity of (1)M to VLDL versus cytotoxicity............... 15

Supplementary Figures

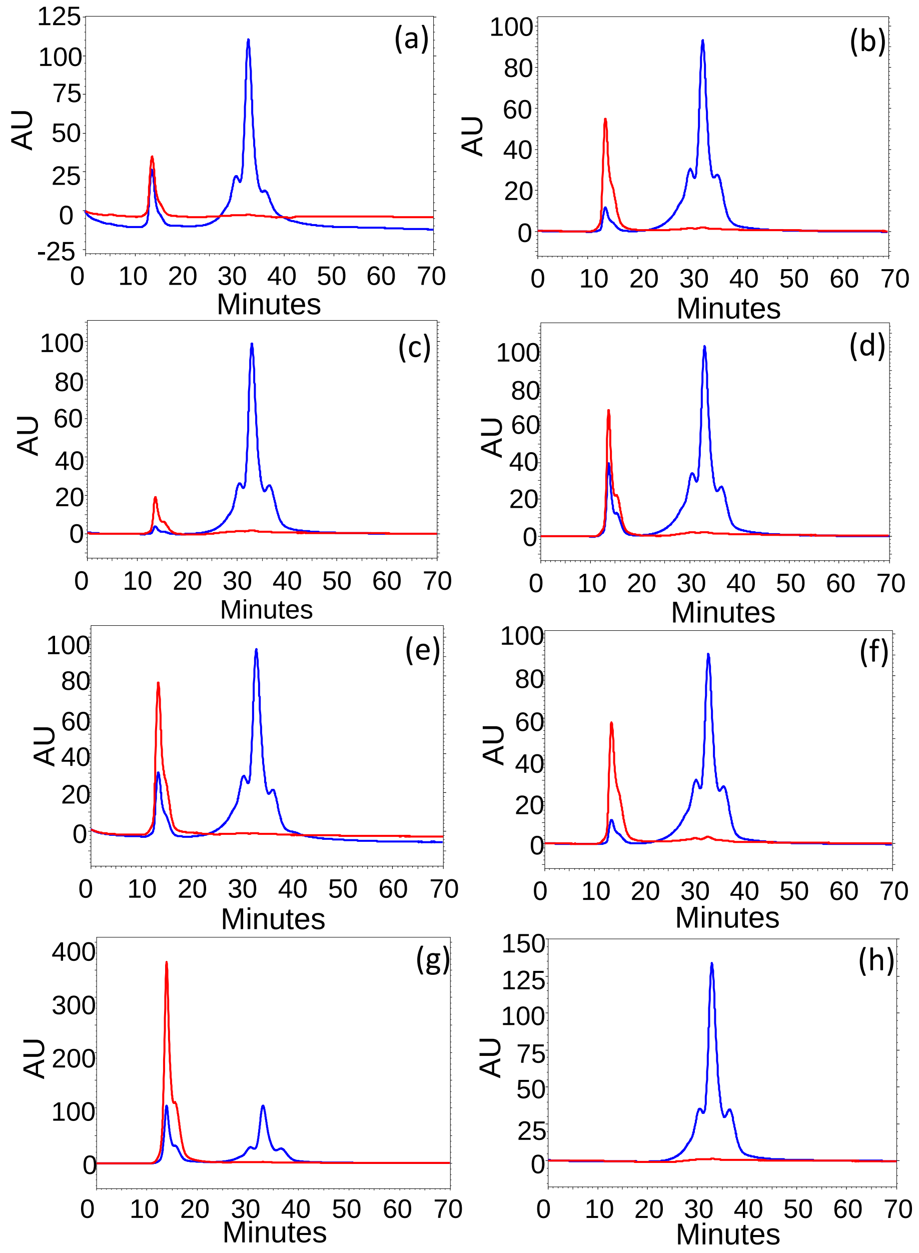

Figure S1. Chromatograms for detection of BSA and the corroles in BSA/corrole conjugates, with the reading set at 280 nm for detection of BSA (blue line) and at 414 nm for detection of corroles (red line): (a) (**2**)Sb, (b) (**2**)Al, (c) (**2**)Ga, (d) (**2**)Mn (e) (**2**)Fe, (f) (**2**)Sn, (g) (**2**)H3 and (h) BSA only. Bioconjugates were formulated as previously discussed (Protocol), and BSA was treated under the same conditions but without the presence of any corrole. Freeze dried samples were re-suspended in PBS and taken to HPLC for analysis. Note that the corroles are present in the fast eluting fraction at about 13 min, which represent much larger molecular weights. That fraction was absent when BSA was treated by the Protocol, but without any corrole derivative.

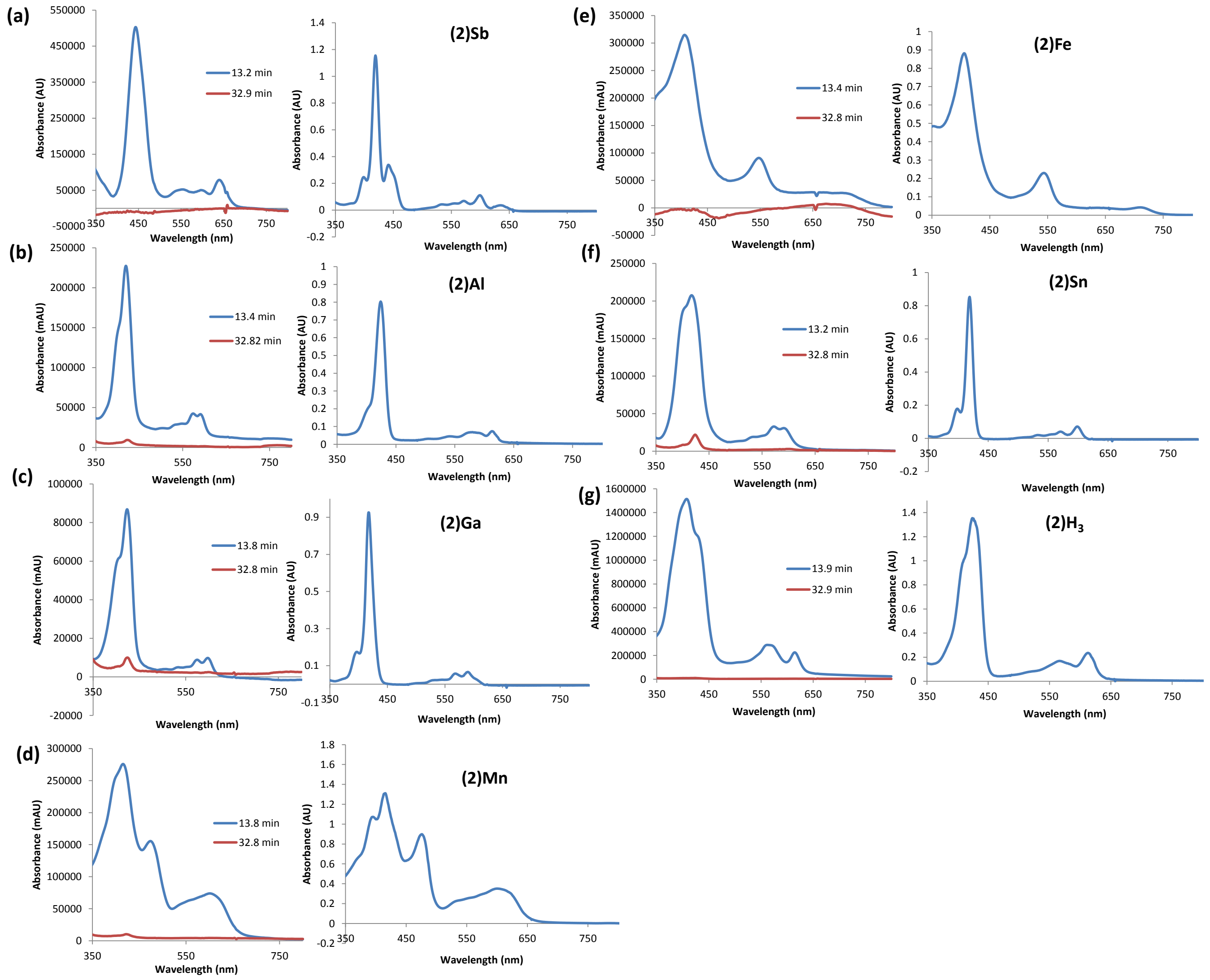

Figure S2. Spectra recorded for (A.) the HPLC-injected corrole/BSA conjugates at different retention times [(a) (**2**)Sb, (b) (**2**)Al, (c) (**2**)Ga, (d) (**2**)Mn (e) (**2**)Fe, (f) (**2**)Sn, (g) (**2**)H3]; and (B.) the same corrole in BSA-free DMSO solution Where are A and B?

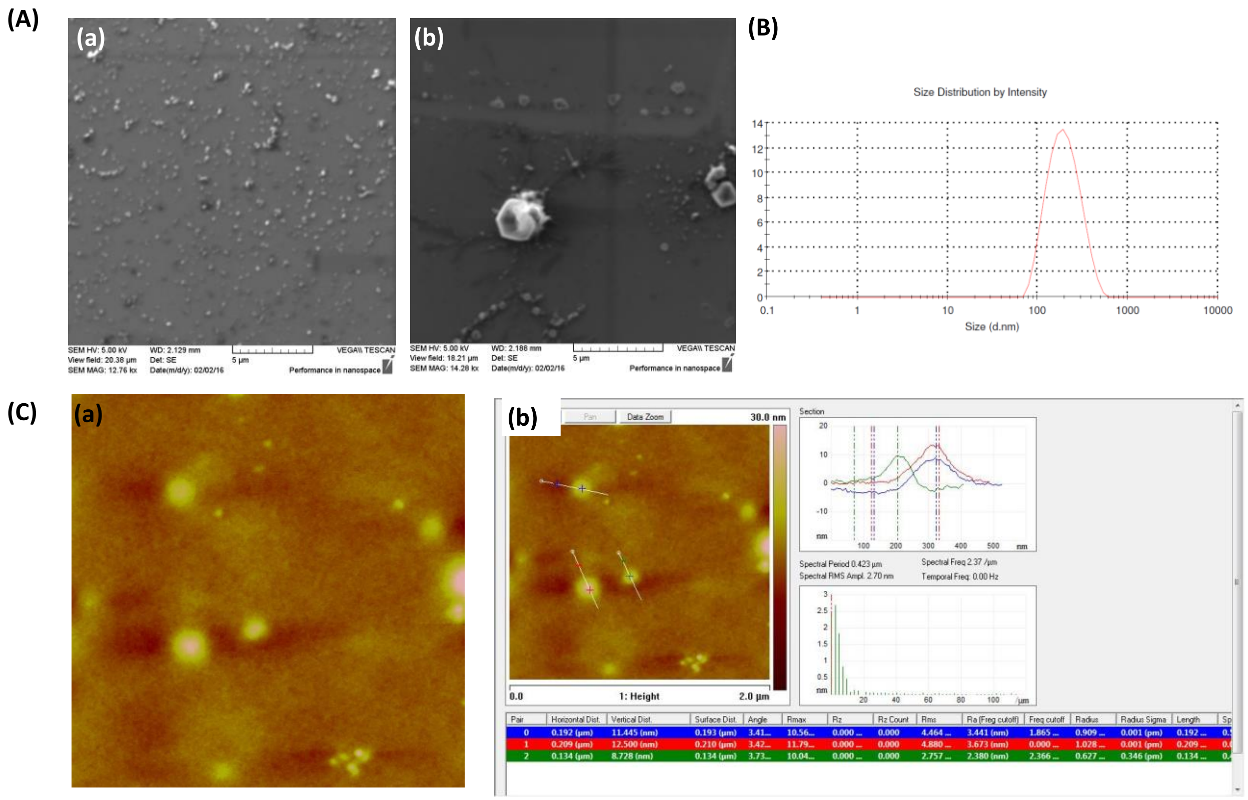

Figure S3. Imaging of nanoparticles and estimation of size by different methods: (A) SEM images of (a) (**2**)Au/BSA isolated NP’s dried on silicon wafer, and (b) treated BSA after centrifugation dried on silicon wafer; (B) DLS distribution plot of isolated and re-suspended in PBS (**2**)Au/BSA NP’s; (C) AFM images of (a) (**2**)Au/BSA isolated NP’s dried on silicon wafer, and (b) estimation of particles distance from silicone surface.

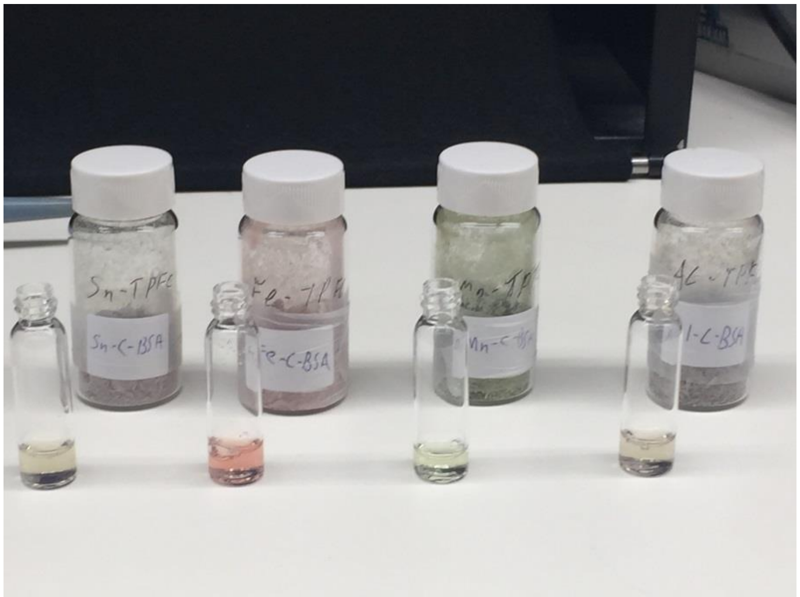

Figure S4. Solubility of formulated biconjugated corrole NP's is indicated in the image above dissolved in PBS pH 7.2 next to the respective NP's in solid form. The corrole BSA bioconjugated NP's are readily soluble in water or buffer (up to 40mg/ml). In contrast TPFC with any metal at its core is highly lipophilic and not soluble in water or buffer ($<10^{-7}$ mg/ml).

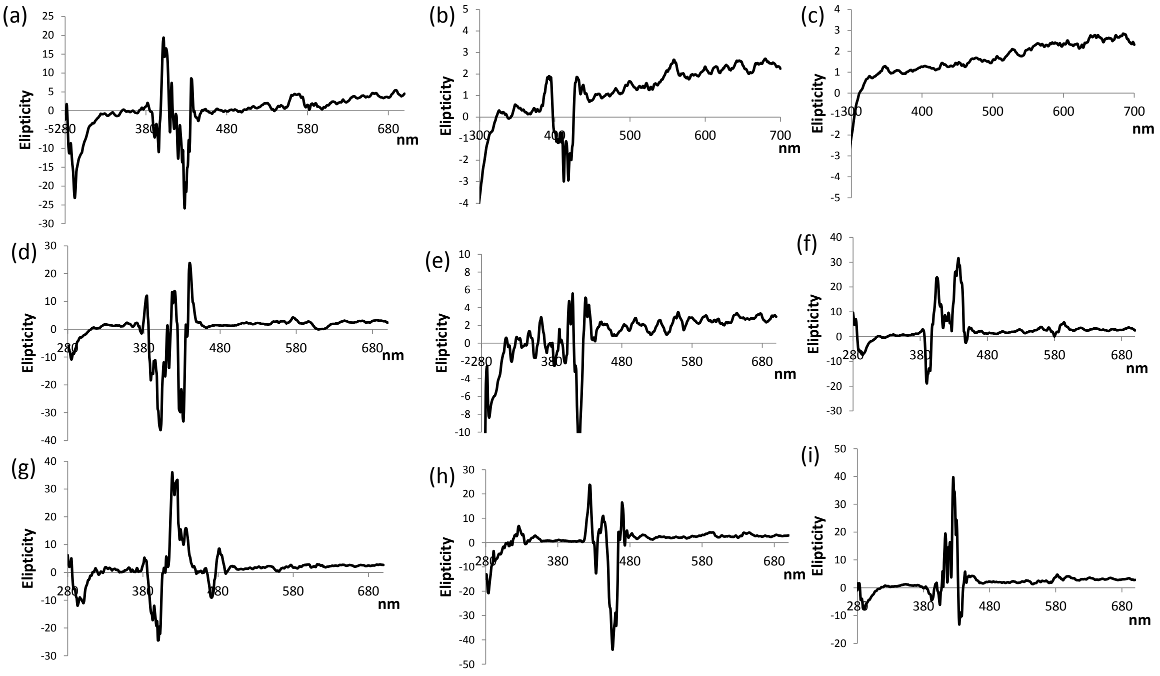

Figure S5. CD spectra of the different corrole NP's: (a.) (**2**)Al, (b.) (**2**)Au, (c.) control (i. e. treated BSA), (d.) (**2**)H3 (free base), (e.) (**2**)Fe, (f.) (**2**)Ga, (g.) (**2**)Mn, (h.) (**2**)Sb and (i.) (**2**)Sn. All other than the control BSA display signals in the visible region, implying interaction between protein and corrole (induced chirality).

(A)

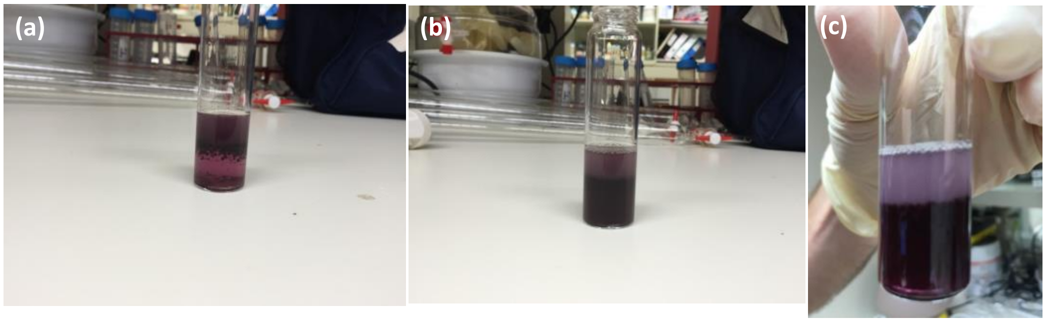

Figure S6. (**2**)Al conjugated to BSA NPs in PBS after extraction. Particles were treated with Triton-X 2%. Triton-X facilitated extraction of corrole from PBS into DCM, shown at different times: a.) 30 sec, b.)1 min and c.) 4 min. DCM fraction was taken directly to run a column using DCM only as an eluent. (**2**)Al was stuck on the top of the column while most unwanted byproducts (e.g., triton-X) were washed down the column. After a few washes with pure DCM, DCM with a few drops of pyridine was added to the column for elution of (**2**)Al. Eluent was evaporated under vacuum and redissolved in C_6D_6 for NMR measurements.

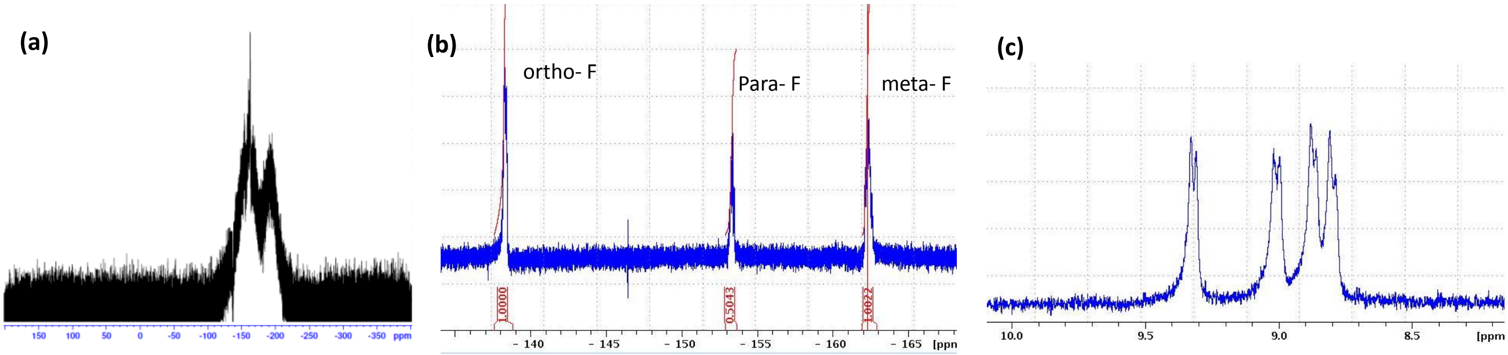

Figure S7. (a) ^{19}F NMR in D_2O (400Mhz) of (**2**)Al/BSA NP's, (b) ^{19}F NMR of (**2**)Al after extraction from NP's (200MHz, in C_6D6) and (c) ^{1}H NMR of (**2**)Al after extraction from NP's (200MHz, in C6D6).

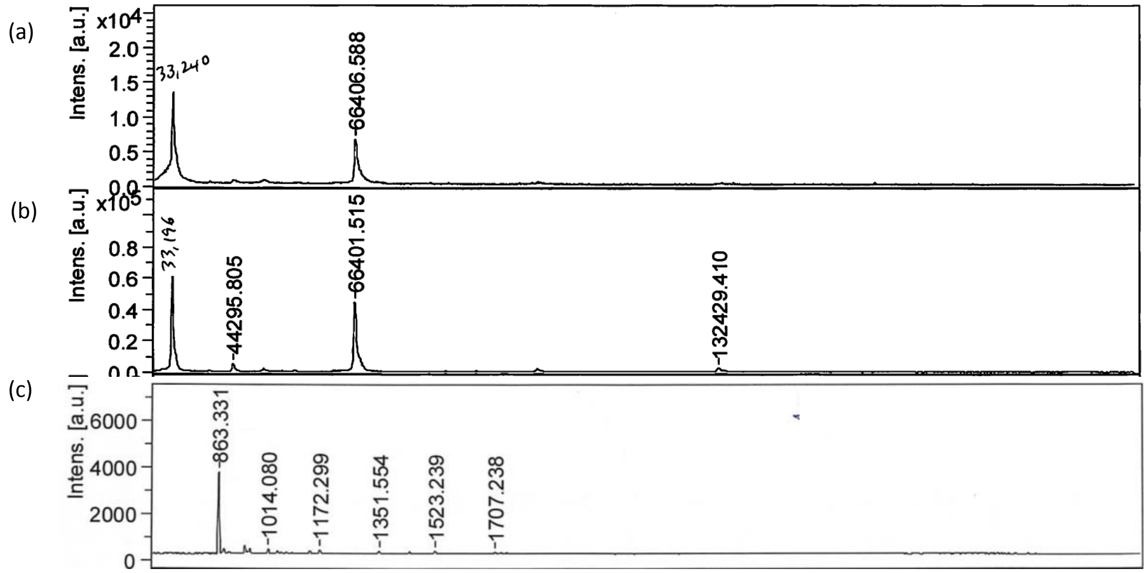

Figure S8. MALDI-mass spec of (**2**)Ga BSA NP's: (a) control (i. e. BSA treated with DMSO without any corrole), (b) (**2**)Ga with BSA NP's, and (c) low region mass spec of (**2**)Ga with BSA NP's.

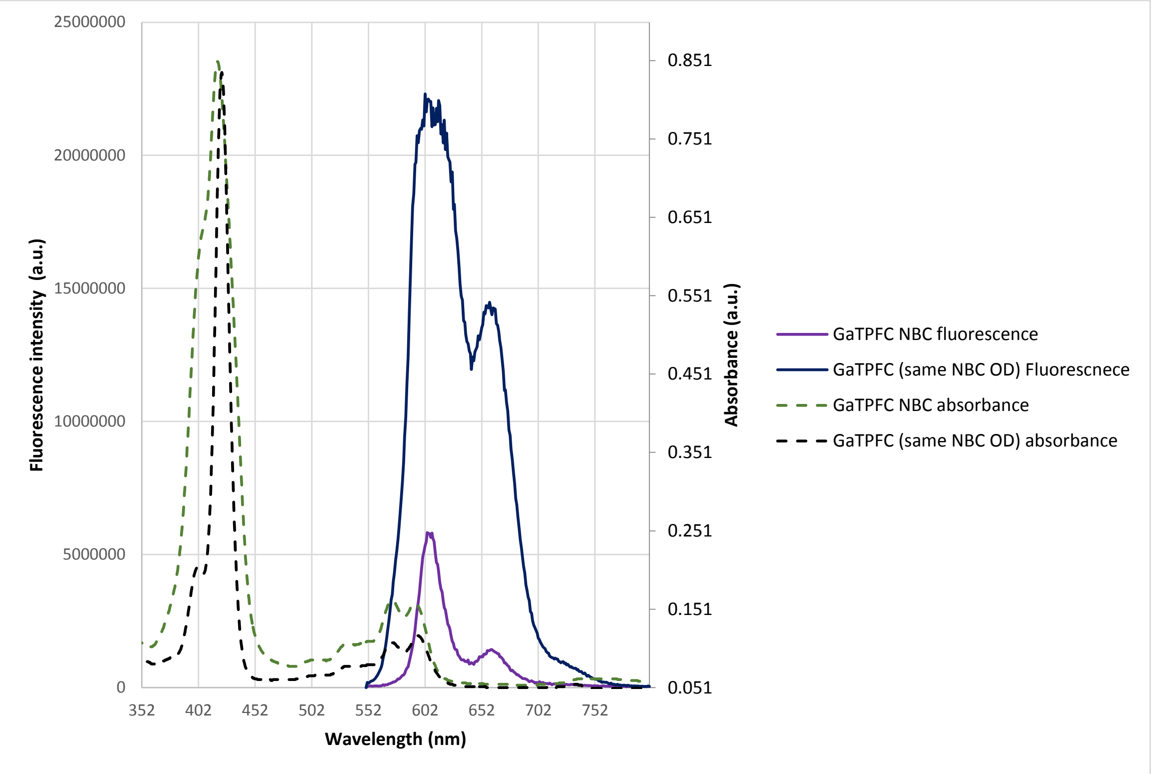

Figure S9. Quenching of the fluorescence of (**2**)Ga upon encapsulation in albumin based NP's as detected by dissolving (**2**)Ga in DMSO to equal absorbance at 420 nm (largely different concentrations) of dialyzed NP's in PBS. Florescence efficiency of detection declines to about 12.6% relative to monomeric (**2**)Ga in DMSO. Estimation of quantum yield of the Np's is 0.048.

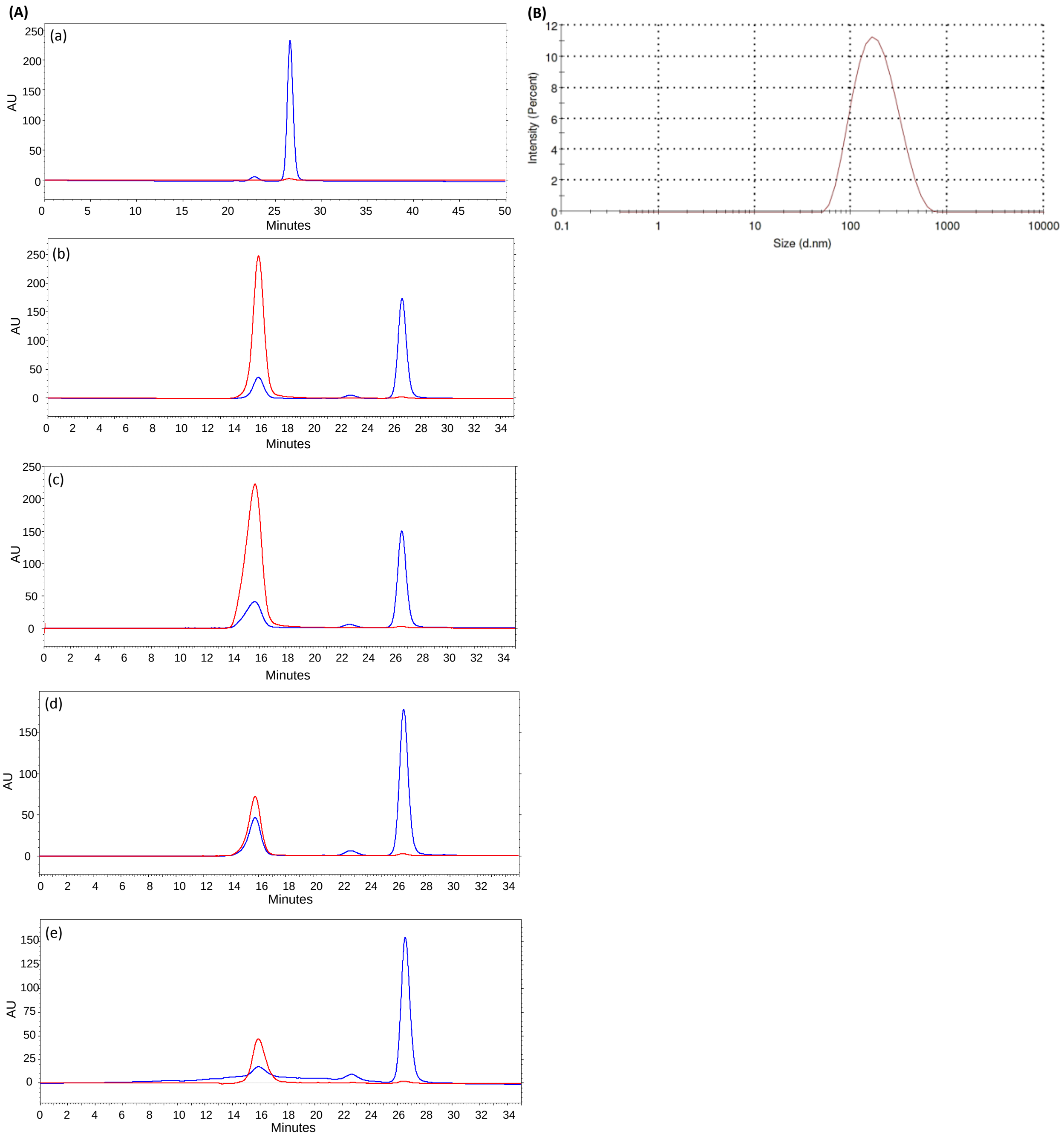

Figure S10. (A) Chromatograms for detection of apo-transferrin (TF) and the corroles in apo-transferrin/corrole conjugates, with the reading set at 280 nm for detection of apo-transferrin (blue line) and at 420 nm for detection of the corroles (red line): (a) apo-transferrin only, (b) (**2**)Ga, (c) (**2**)Sn, (d) (**2**)Mn and (e) (**2**)Au; (B) DLS analysis of (**2**)Au/TF NP's, size of observed particles was 173.3 ± 64.09 nm;

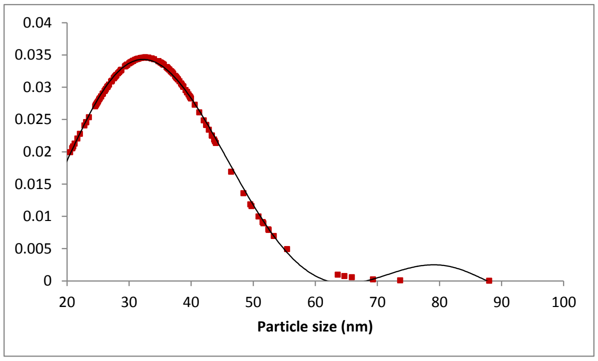

Figure S11. Particle size distribution as analyzed from 12 separate fields in two distinct samples of (**2**)Au/HSA NPs (imaged using Cryo-TEM).

Table S1. IC_{50} values of (**2**)M series in DMSO(aq.) 0.1% recorded for different cancer cell lines.

	***(2)*Ga**	***(2)*Au**	***(2)*Sb**	***(2)*Fe**	***(2)*Mn**	***(2)*Al**
**DU145**	156.6	> 400	> 400	297.9	> 400	> 400
**SK-MEL-28**	171.5	> 400	> 400	116.4	> 400	> 400
**MDA-MB-231**	177.1	> 400	296.5	115.7	129.1	> 400
**OVCAR-3**	110.5	> 400	> 400	352.6	> 400	> 400

Table S2. Asymmetric oxidation of thioanisole by H_2O_2 in solutions that contained either HSA, BSA, or NPs composed of HSA/BSA with Fe(III)/Mn(III)corroles.

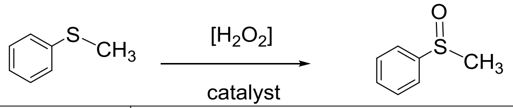

Catalyst	Albumin source yield %; major enantiomer; ee %	
	HSA	BSA
HSA-blank	12; R; 1	-
BSA-blank	-	4; R; 1
(2)Mn	13; R; 11	77; R; 7
(2)Mn	21; R; 2;	-
(2)Mn	1; R; 2	-
(2)Fe	70; R; 15	96; R; 1
(2)Fe	4; R; 1	-
(2)Fe	14; R; 16	-

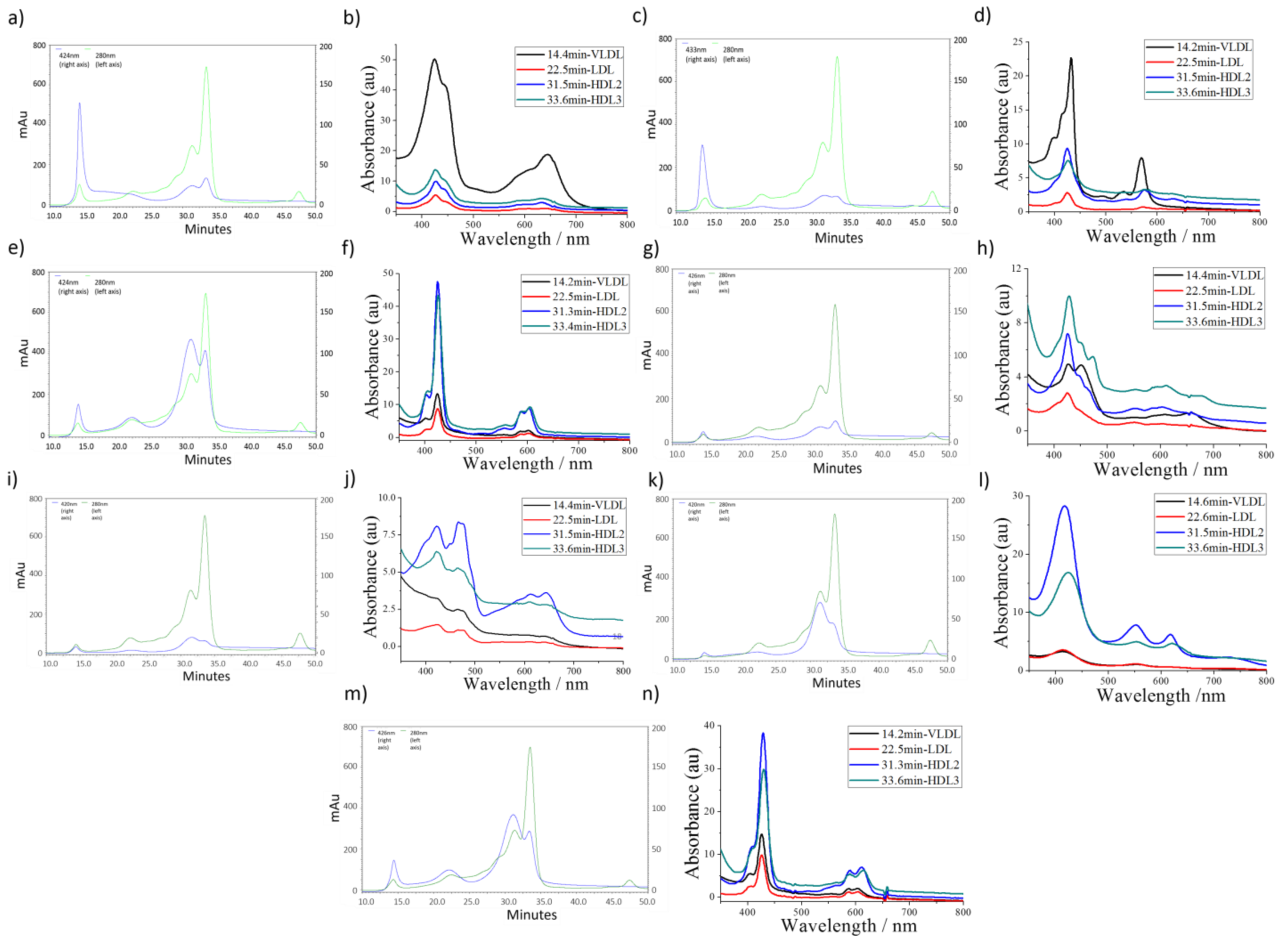

Figure S12. HPLC traces of human serum incubated with the corresponding corroles (**1**)H_3 (a & b) and the corresponding (**1**)Au (c & d), (**1**)Al (e & f), (**1**)Sb (g & h), (**1**)Mn (i & j), (**1**)Fe (k & l), (**1**)Ga (m & n) complexes, together with UV-vis spectra of the eluted fractions. The green and blue traces are chromatograms obtained by following changes at 280 nm (to determine protein elution) and at the λ_{max} of the eluting corrole (about 420 nm), respectively.

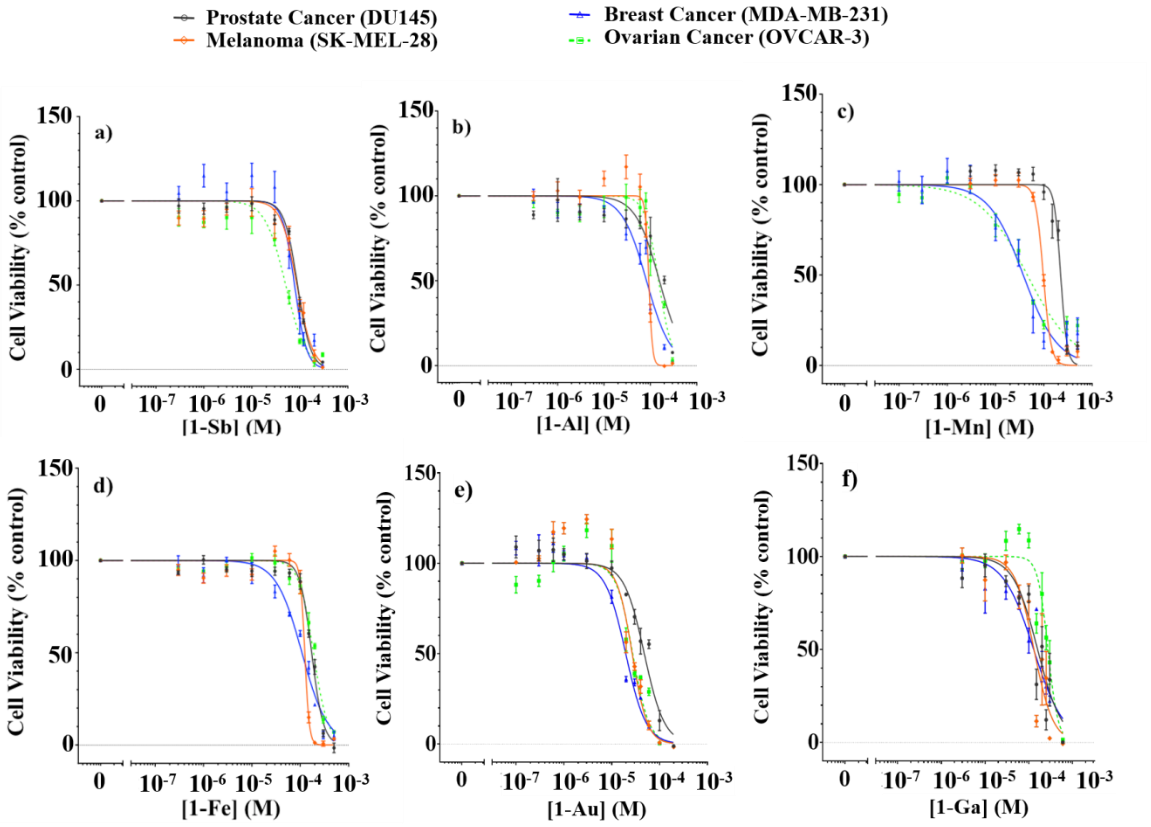

Figure S13. Cytotoxicity graphs of (**1**)M series (M = a) Sb, b) Al, c) Mn, d) Fe, e) Au, f) Ga, against four NCI-60 human cancer cell lines.

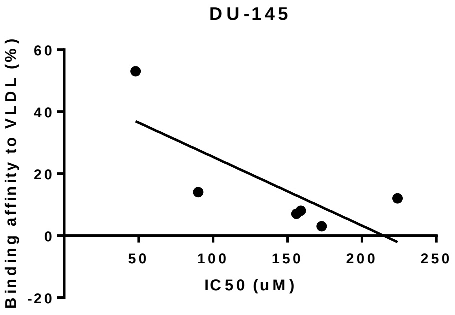

Pearson r	
r	-0.7538
95% confidence interval	-0.9712 to 0.1492
R square	0.5681
P value	
P (two-tailed)	0.0835
P value summary	ns
Significant? (alpha = 0.05)	No

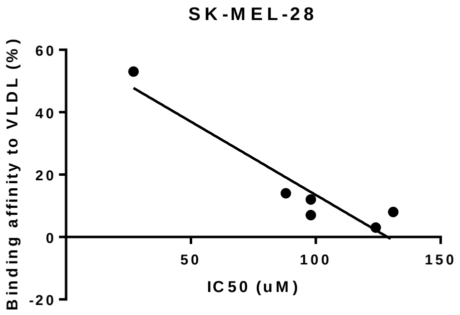

Pearson r	
r	-0.9402
95% confidence interval	-0.9936 to -0.5427
R square	0.8840
P value	
P (two-tailed)	0.0053
P value summary	**
Significant? (alpha = 0.05)	Yes

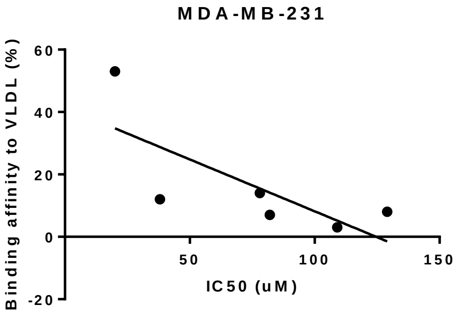

Pearson r	
r	-0.7433
95% confidence interval	-0.9698 to 0.1722
R square	0.5525
P value	
P (two-tailed)	0.0904
P value summary	ns
Significant? (alpha = 0.05)	No

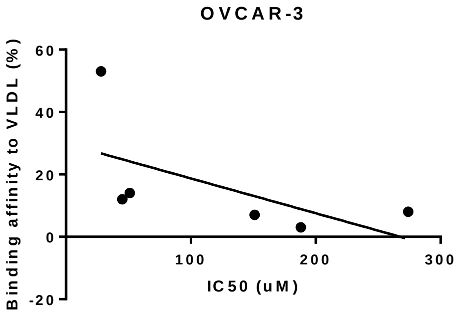

Pearson r	
r	-0.5927
95% confidence interval	-0.9482 to 0.4220
R square	0.3512
P value	
P (two-tailed)	0.2151
P value summary	ns
Significant? (alpha = 0.05)	No

Figure S14. Percentage binding affinity of (**1**)M to VLDL versus cytotoxicity for each of the four cell lines studied – a) DU145, b) SK-MEL-28, c) MDA-MB-231, and d) OVCAR-3. For each plot, each data point corresponds to (**1**)M. The data were fitted with a linear function.
